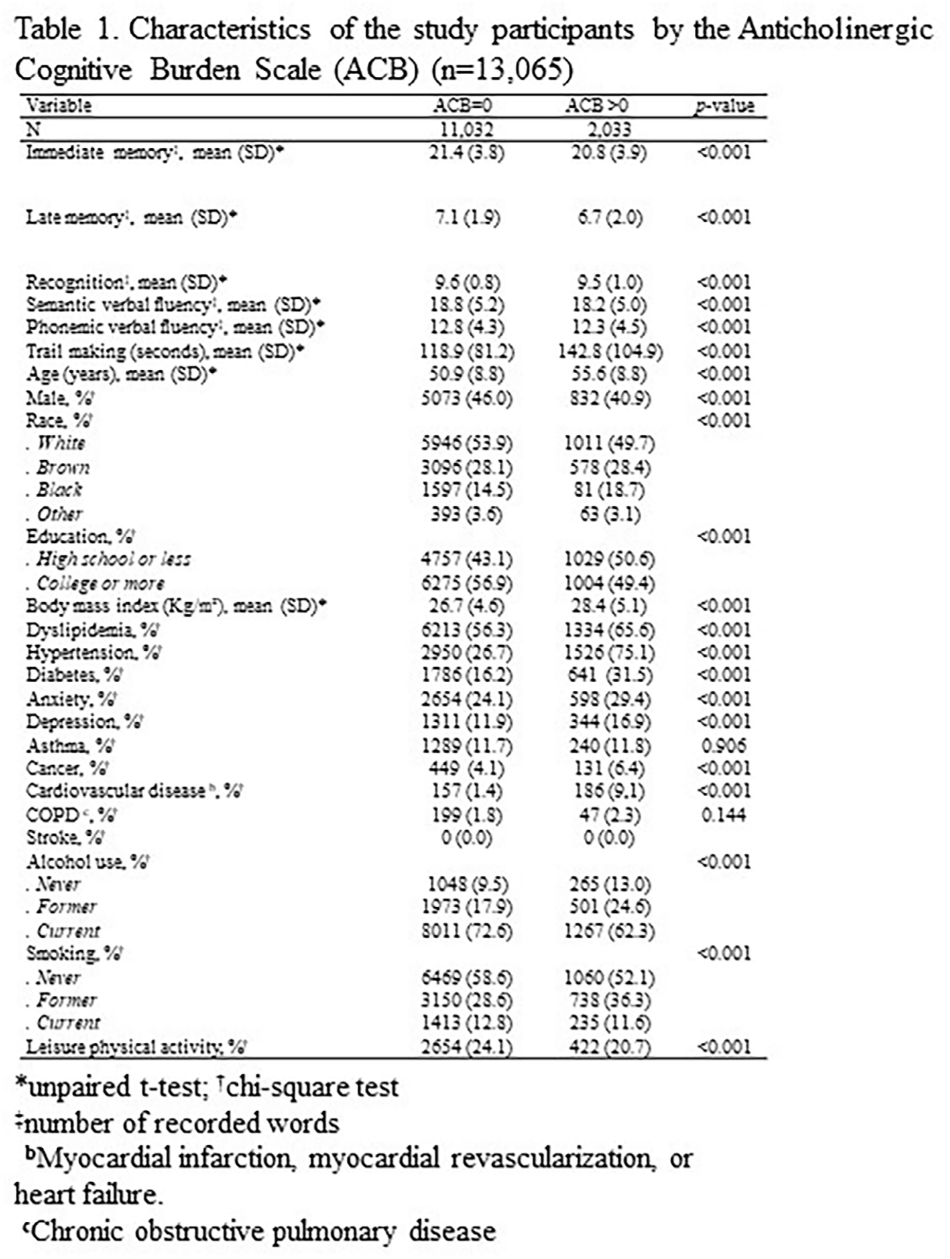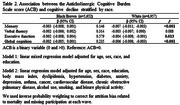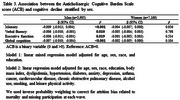# Racial and Sex differences in the association between anticholinergic burden and cognitive decline: longitudinal findigns from the Elsa‐Brasil Study

**DOI:** 10.1002/alz70860_101999

**Published:** 2025-12-23

**Authors:** Adriana Nancy Medeiros dos Santos, Natalia Gomes Goncalves, Alessandra C. Goulart, Maria Carmen Viana, Paulo A Lotufo, Isabela M. Benseñor, Claudia Kimie Suemoto

**Affiliations:** ^1^ University of São Paulo Medical School, São Paulo, Brazil; ^2^ University of São Paulo Medical School, São Paulo, SP, Brazil; ^3^ University of São Paulo M, Sao Paulo, Brazil; ^4^ University of São Paulo Medical School, SÃO PAULO, Brazil; ^5^ University of Sao Paulo, Sao Paulo, Brazil, Brazil; ^6^ University of São Paulo Medical School, São Paulo, São Paulo, Brazil

## Abstract

**Background:**

The impact of anticholinergic burden on cognition is a debated topic, with evidence linking it to cognitive decline in previous studies. However, few studies examine how these associations may differ by race and gender, particularly in racially diverse countries like Brazil. The Longitudinal Study of Adult Health (ELSA‐Brasil) offers a unique opportunity to investigate these disparities, as it includes a large and diverse population sample. We aimed to explore racial and sex differences in the longitudinal relationship between Anticholinergic Cognitive Burden (ACB) and cognitive decline over eight years of follow‐up in the ELSA‐Brasil study.

**Method:**

The ELSA‐Brasil is a multicenter, prospective cohort study conducted across six Brazilian cities. Participants were civil servants aged 35‐74 years at baseline. Individuals with missing data on medication use, cognitive tests, or covariates were excluded. ACB was measured at baseline using the ACB scale, and cognitive performance was assessed with tests of immediate and delayed word recall, word recognition, verbal fluency, and the trail‐making version B, performed across three study waves (2008‐2010, 2012‐2014, and 2017‐2019). Linear mixed‐effects models were used to investigate the association between ACB and cognitive decline.

**Result:**

Of the 15,105 participants recruited, 2,040 were excluded, leaving 13,065 for analysis. The mean age was 51.7±9.0 years, with 55% women and 53% white participants (Table 1). ACB burden was linked to cognitive decline in global cognition (β = ‐0.006, *p* <0.001), memory (β = ‐0.007, *p* <0.001), and executive function (β = ‐0.006, *p* <0.001) in white participants (Table 2). In men, ACB was associated with declines in global cognition (β = ‐0.007, *p* <0.001), memory (β = ‐0.009, *p* <0.001), executive function (β = ‐0.006, *p* = 0.019), and verbal fluency (β = ‐0.006, *p* = 0.010) (Table 3). There was no association between ACB and cognitive decline for black/brown participants and women.

**Conclusion:**

Higher ACB was differently linked to cognitive decline by race and sex. These associations were more pronounced in white and male participants, emphasizing the importance of considering racial and sex differences in the effects of anticholinergic medications on cognitive health.